# Numerical simulation on energy transfer enhancement of a Williamson ferrofluid subjected to thermal radiation and a magnetic field using hybrid ultrafine particles

**DOI:** 10.1038/s41598-023-29707-5

**Published:** 2023-02-23

**Authors:** Mohammed Z. Swalmeh, Firas A. Alwawi, Muhammad Salman Kausar, Mohd Asrul Hery Ibrahim, Abdulkareem Saleh Hamarsheh, Ibrahim Mohammed Sulaiman, Aliyu Muhammed Awwal, Nuttapol Pakkaranang, Bancha Panyanak

**Affiliations:** 1 Faculty of Arts and Sciences, Aqaba University of Technology, Aqaba, 77110 Jordan; 2 Department of Mathematics, College of Sciences and Humanities in Al-Kharj, Prince Sattam Bin Abdulaziz University, Al-Kharj, 11942 Saudi Arabia; 3 Faculty of Informatics and Computing, Universiti Sultan Zainal Abidin (Kampus Gong Badak), 21300 Kuala Terengganu, Terengganu Malaysia; 4 Faculty of Entrepreneurship and Business, Universiti Malaysia Kelantan, 16100 Kelantan, Malaysia; 5 School of Quantitative Sciences, Institute of Strategic Industrial Decision Modelling, Universiti Utara Malaysia, Sintok, 06010 Kedah, Malaysia; 6 Department of Mathematics, Faculty of Science, Gombe State University (GSU), Gombe, Nigeria; 7 GSU-Mathematics for Innovative Research Group, Gombe State University (GSU), Gombe, Nigeria; 8 Mathematics and Computing Science Program, Faculty of Science and Technology, Phetchabun Rajabhat University, Phetchabun, 67000 Thailand; 9 Research Group in Mathematics and Applied Mathematics, Department of Mathematics, Faculty of Science, Chiang Mai University, Chiang Mai, 50200 Thailand; 10 Department of Mathematics, Faculty of Science, Data Science Research Center, Chiang Mai University, Chiang Mai, 50200 Thailand

**Keywords:** Applied mathematics, Computational science, Scientific data

## Abstract

In this numerical investigation, completely developed laminar convective heat transfer characteristics of a Williamson hybrid ferronanofluid over a cylindrical surface are reported. This new model in 2D is engaged to examine the effects of the magnetic field, thermal radiation factor, volume fraction of ultrafine particles, and Weissenberg number with the help of the Keller box method. The numerical calculations are implemented at a magnetic parameter range of 0.4 to 0.8, volume fraction range of 0.0 to 0.1, and a Weissenberg number range of 0.1 to 0.8. The numerical outcomes concluded that the velocity increases when the thermal radiation parameter and the volume fraction of a nanoparticle are increased, but inverse impacts are obtained for the magnetic parameter and the Weissenberg number. The rate of energy transport increases with increasing thermal radiation and volume fraction, while it declines with increasing the magnetic parameter and Weissenberg number. The drag force shows a positive relationship with the thermal radiation parameter and has an opposite relationship with the Weissenberg number and the magnetic parameter. Furthermore, even when the magnetic field, thermal radiation, volume fraction, and Weissenberg number are all present, the heat transfer rate of Williamson hybrid ferronanofluid is greater than that of mono Williamson ferronanofluid.

## Introduction

The presence of nanoparticles suspended in a base fluid such as ethylene glycol, water, or oil is known as nanofluid. In most cases, these nanoparticles are composed of carbon nanotubes, oxides, and metals. Nanofluids offer novel features that make them beneficial in a wide variety of applications of heat transfer; some of these applications include heat exchangers, heat pipes, solar collectors, microelectronics, and fuel cells^1–6^. To figure out how these nanoparticles affect a host fluid’s heat transfer capabilities, it is important to look at their thermophysical properties, especially when it comes to applications that involve heat transfer. Assessing thermal conductivity is among the most important thermophysical parameters. Nanofluids exhibit improved thermal conductivity and thus a higher heat transfer rate compared to standard fluids. Many studies have been devoted to exploring the factors affecting the thermal conductivity of nanoparticles^7–11^. They found that nanofluids’ thermal conductivity depends on some fundamental features, which include shape and size, and the volume fraction of nanoparticles.

Even if mono-nanofluids offer a great deal of promise to quench the ever-increasing thirst for increased thermal efficiency, researchers are nevertheless actively exploring new kinds of fluids. One of these types of modern fluids that has begun to emerge recently is the new generation of nanofluids, or what is known as hybrid nanofluids^12^. In comparison to mono-nanofluids, hybrid nanofluids demonstrate a considerable improvement in their thermal efficiency^13,14^. These fluids are produced by dispersing two or more varieties of nanoparticles or composite nanostructures in a base fluid. This denotes the existence of a homogenous mixture that possesses the physicochemical features of a number of different substances, the likes of which are extremely unlikely to be found in a single entity. Mono-nanofluids have gradually been replaced by hybrid nanofluids in a variety of heat transfer applications, including generator cooling, thermal storage, biomedical applications, electronic cooling, automobile radiators, lubrication, welding, nuclear system cooling, coolant in machining, solar heating, cooling, and heating in buildings, drug reduction, refrigeration, etc., due to the advantageous properties shown by these new generations of fluids, such as greater chemical stability and higher thermal efficiency, both of which contribute to the improved performance of hybrid nanofluids in industrial settings^15–17^. Over the course of the last few years, many studies have been carried out to numerically investigate the characteristics of the flow of hybrid nanofluids^18–22^. This study employs engine-oil-based Fe_3_O_4_ as a ferro-mono nanofluid, which is distinguished by its strong response to magnetic field influence. Ferro-nanofluid is typically composed of tiny magnetic nanosolids suspended in a host fluid. These tiny magnetic particles include hematite, ferrite, magnetite, iron, iron oxide, etc.^23–25^. The importance of these fluids lies in their many engineering applications^26–29^. In order to support the thermal properties of this mono-ferronanofluid, ultrafine aluminum oxide particles Al_2_O_3_ are adopted.

Heat transfer via thermal radiation is of greater importance when it comes to aerospace applications, high operating temperatures, and power engineering. Thermal radiation also has a critical role in controlling heat transfer, especially in the polymer processing industry. In addition, the majority of solar energy-based industries, such as solar energy collectors, are applications of heat transfer via thermal radiation in the presence of natural convection. Several scholars have studied the effect of thermal radiation on a variety of fluid flow patterns in the case of free convection. KA^30^ presented a numerical study of viscous incompressible fluid flow past a vertical cylinder in a porous medium with thermal radiation impacts. Later, Sheikholeslami et al.^31^ numerically simulated the free convection and characteristics of the flow of nanoliquids with a thermal radiation effect. Akram et al.^32^ analyzed the magnetic hydrodynamics free convection boundary layer flow of nanoliquid around a cylindrical object in the presence of thermal radiation and heat generation.

Many scientists and researchers are interested in the flow and heat transport of moving liquids under the influence of the magnetic hydrodynamics (MHD) field because of the wide range of applications it has in manufacturing and mechanics, optical gratings, cooling systems for thermonuclear fusion power plants, fuel and gas technologies, mechanical flow, hydraulic pumps, etc.^33–35^, on the other hand, many mathematical models have appeared that attempt to simulate the behavior of non-Newtonian fluids, among the most realistic of which is the model presented by Williamson^36^ in 1929. He predicted the flow properties of shear-thinning liquids. Recently, several studies have utilized the Williamson model in the presence of a magnetic field. In their study, Asjad et al.^37^ examined MHD Williamson fluid flow in the company of thermal radiation and microorganisms. They discovered that as the magnetic parameter was increased, the fluid velocity decreased. Almaneea^38^ provided insight into the effect of hybrid nanosolids with homogenous/heterogenous chemical reaction on magneto-Williamson fluid. Dadheech et al.^39^ described MHD flow along with Cattaneo–Christov heat flux. They looked into the thermo-physical characteristics of the Williamson fluid with slip effects. Bhatti et al.^40^ reported on the gyrotactic microorganism porous media effects of nanoparticles on MHD Williamson fluid flow connecting two rotating circular plates in an embedded system. Akbar et al.^41^ discussed the performance of Williamson fluid past a porous stretching surface with Dufour/Soret and mixed convection MHD effects. A study of non-Fourier Williamson hybrid nanofluid flow under the influence of Hall, ion slip currents, and a non-uniform magnetic field was done by Salmi et al.^42^. They obtained a numerical solution with the help of the finite element method and pointed out that fluid motion is reduced for greater values of the Weissenberg number. Mishra et al.^43^ illustrated the related influences of thermal conductivity and non-Darcian porous media characteristics on micropolar and Williamson fluid MHD flow over a stretching surface. Bijarchi et al.^44^ investigated the ferrofluid droplets influence of non-uniform magnetic field analysis over large/small computational domains with non‑linear magnetic permeability. Also see the following recent references.^19,20,45–48^.

Based on the above-mentioned studies, the main target of this study is to consider the problem of heat transfer with radiation impacts on electro-conductive Williamson hybrid nanofluid. Also, the current study is interested in the effects of magnetic fields over a horizontal circular cylinder, subject to constant heat flux boundary conditions. Under various relevant parameters, the two different types of nanoparticles suspended in engine oil-based fluid, alumina and iron oxide, are used as hybrid nanosolids. The expanded Tiwari and Das model is converted into a non-dimensional partial differential equations (PDEs) using suitable transformations and variables. Furthermore, the numerical solutions for these PDEs are obtained by the Keller box method. The impacts of related parameters on local skin friction, local Nusselt number, temperature, and velocity profiles are showcased via graphs and tables.

## Mathematical formulations

**Figure 1:**
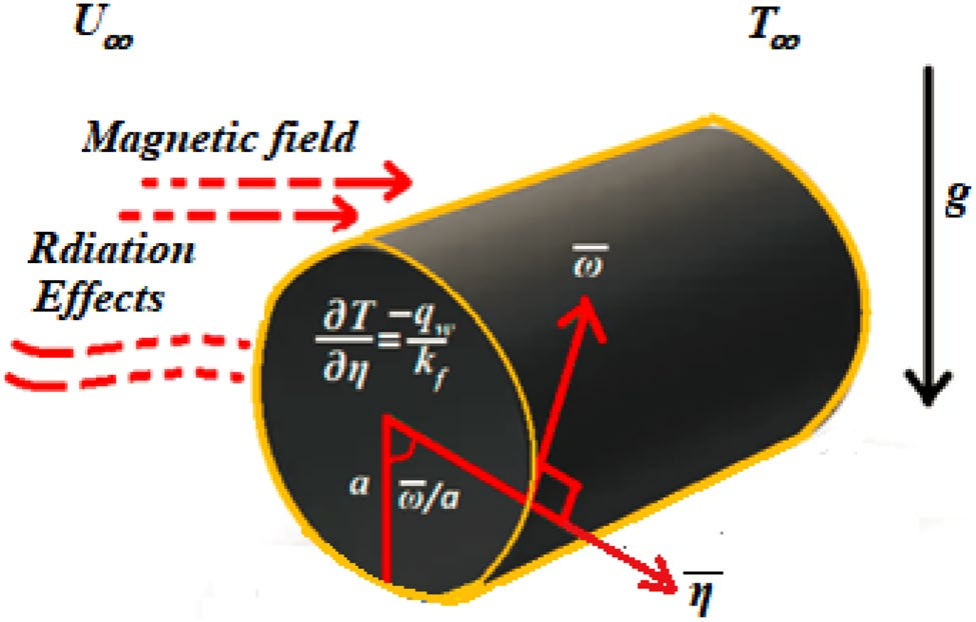
MHD Williamson hybrid ferronanofluid physical model.

In this section, we begin by establishing the mathematical formulations for the problem of two-dimensional steady laminar natural convection flow of Williamson ferrofluid over a circular cylinder under the Boussinesq approximation. A hybrid composition of alumina and iron oxide is suspended in the host fluid, which is influenced by a transverse magnetic field and thermal radiation. On the other hand, the constant heat flux boundary conditions have been taken into consideration. It is noteworthy that the goal of choosing a hybrid ferronanofluid in this research is to understand the thermal heat transfer enhancements when composing $\chi_{{{\text{Al}}_{2} {\text{O}}_{3} }}$ with $\chi_{{{\text{Fe}}_{3} {\text{O}}_{4} }}$. Assume that *ω* measures the circumference of the circular cylinder surface and *η* is perpendicular to it, as well as that *g*, *T*_*∞*_, and* U*_*∞*_ are gravity, ambient temperature, and velocity, respectively. These assumptions are represented in Fig. 1.

The constituent equations of the Williamson fluid model are shown as (see^49,50^):

1 $$\begin{array}{*{20}l} {S = - pI + \lambda ,} \hfill \\ {\lambda = \left( {\mu_{\infty } + \frac{{\mu_{0} - \mu_{\infty } }}{1 - \Gamma \aleph }} \right)A_{1} ,} \hfill \\ \end{array}$$

where $p,I,\,\,{\text{and}}\,\lambda$ are pressure, identity vector, and extra stress tensor, respectively. $\Gamma$ is the time constant. $\mu_{\infty } ,\,\,{\text{and}}\,\mu_{0}$ are the limiting viscosities at infinity and zero shear rate. $A_{1}$ is called the first Rivlin-Erickson tensor and $\aleph$ is presented as follows:

2 $$\begin{gathered} \aleph = \sqrt {0.5\,\pi } , \hfill \\ \pi = trac({\text{A}}_{1}^{2} ). \hfill \\ \end{gathered}$$

The case $,\mu_{\infty } = 0,\Gamma \aleph < 0,$ is taken, so the Eq. (1) can be expressed as:

3 $$\lambda = \left( {\frac{{\mu_{0} }}{1 - \Gamma \aleph }} \right)A_{1} .$$

Consequently, the formula can be rewritten via binomial expansion as:

4 $$\lambda = \mu_{0} \left( {1 + \Gamma \aleph } \right)A_{1} .$$

Depending on the above assumptions, the continuity, momentum, and energy vectorial equations are deduced as follows:

5 $$\vec{\nabla } \cdot \vec{V} = 0,$$

6 $$\begin{array}{*{20}l} \left( {\vec{V} \cdot \vec{\nabla }} \right)\vec{V} = - \frac{1}{{\rho_{F - Hnf} }}\vec{\nabla }\vec{P} + \left( {\frac{{\mu_{F - Hnf} }}{{\rho_{F - Hnf} }}} \right)\vec{\nabla }^{2} \vec{V} + \frac{{\sqrt 2 \vec{v}\Gamma }}{{\rho_{F - Hnf} }}\left( {\frac{{\partial^{2} \vec{u}}}{{\partial \vec{\omega }^{2} }}\frac{{\partial \vec{u}}}{{\partial \vec{\omega }}}} \right) \hfill \\ \frac{{\beta_{f - Hnf} }}{{\rho_{F - Hnf} }}\hat{g}\left( {T - T_{\infty } } \right) - \frac{{\sigma_{F - Hnf} }}{{\rho_{F - Hnf} }}B_{0}^{2} \vec{u}, \hfill \\ \end{array}$$

7 $$\left( {\vec{V} \cdot \vec{\nabla }} \right)T = \alpha_{F - Hnf} \vec{\nabla }^{2} T - \frac{1}{{(\rho c_{p} )_{F - Hnf} }}\frac{{\partial Q_{R} }}{{\partial \vec{\eta }}},$$

expressions $\vec{g}$, $\vec{P}$, $\vec{V}$, and $\hat{\nabla }^{2}$ are gravitational, pressure, velocity vectors, and Laplacian operator, respectively. $\omega ,\,{\text{and}}\,\eta$ are velocity components.$\mu ,\rho ,\beta ,\sigma ,\,{\text{and}}\,T$ are dynamic viscosity, density, thermal expansion, electrical conductivity, and temperature, respectively. $(c_{p} ),\,\,{\text{and}}\,Q_{R}$ are heat capacity and Rosseland diffusion approximation. The subscript $F - Hnf$ denotes the hybrid ferronanofluid. The vector $\vec{g}$ can be split into $g_{\omega } = g\sin (\omega /a),$ and $g_{\eta } = g\cos (\omega /a)$, so the momentum equation can be presented in $\omega - \eta$ directions. Furthermore, by utilizing the boundary layer approximations $Gr \to \infty$, getting $(1/Gr) \to 0$ and $- (\partial P/\partial \omega ) = 0$ (natural convection flow case), all the terms that include $(1/Gr)$ can be neglected. Here $Gr$ is the Grashof number, which is given by $Gr = g\beta_{f} (aq_{w} /k_{f} )\frac{{a^{3} }}{{\nu_{f}^{2} }}$.

Depending on that, the above dimensional governing Eqs. (5)–(7) become:

8 $$\frac{{\partial \vec{u}}}{{\partial \vec{\omega }}} + \frac{{\partial \vec{v}}}{{\partial \vec{\eta }}} = 0,$$

9 $$\begin{aligned} \left( {\vec{u}\frac{{\partial \vec{u}}}{{\partial \vec{\omega }}} + \overline{v}\frac{{\partial \vec{u}}}{{\partial \vec{\eta }}}} \right) = & \frac{\sqrt 2 v\Gamma }{{\rho_{F - Hnf} }}\left( {\frac{{\partial^{2} \vec{u}}}{{\partial \vec{\eta }^{2} }}\frac{{\partial \vec{u}}}{{\partial \vec{\eta }}}} \right) + \frac{{\mu_{F - Hnf} }}{{\rho_{F - Hnf} }}\left( {\frac{{\partial^{2} \vec{u}}}{{\partial \vec{\omega }^{2} }} + \frac{{\partial^{2} \vec{u}}}{{\partial \vec{\eta }^{2} }}} \right) \\ & + \beta_{F - hnf} g(T - T_{\infty } )\sin \left( {\frac{{\vec{\omega }}}{a}} \right) - \frac{{\sigma_{F - Hnf} }}{{\rho_{F - Hnf} }}B_{0}^{2} \vec{u}, \\ \end{aligned}$$

10 $$\vec{u}\frac{\partial T}{{\partial \vec{\omega }}} + \vec{v}\frac{\partial T}{{\partial \vec{\eta }}} = \alpha_{F - Hnf} \left( {\frac{{\partial^{2} T}}{{\partial \vec{\omega }^{2} }} + \frac{{\partial^{2} T}}{{\partial \vec{\eta }^{2} }}} \right) - \frac{1}{{(\rho c_{p} )_{F - Hnf} }}\frac{{\partial Q_{R} }}{{\partial \vec{\eta }}}.$$

The constant heat flux boundary condition are (see^51^):

$$\vec{u} = \vec{v} = 0,\,\frac{\partial T}{{\partial \vec{\eta }}} = \frac{{ - q_{w} }}{{k_{f} }}\,\,{\text{at}}\,\,\,\,\,\,\vec{\eta } = 0,$$

11 $$\vec{u},\vec{v} \to 0,T \to T_{\infty } ,\,\,\,{\text{at}}\,\,\,\,\,\vec{\eta } \to \infty,$$

where $\overline{u}$ and $\overline{v}$ are velocity components along $\omega - \eta$ orientation. Carrying out the transformation from dimensional to non-dimensional equations, the non-dimensional variables are defined as: (see^52,53^):

12 $$\omega = \left( {\frac{{\vec{\omega }}}{a}} \right),\eta = Gr^{1/5} \left( {\frac{{\vec{\eta }}}{a}} \right),\,\,u = \left( {\frac{a}{{v_{f} }}} \right)Gr^{ - 2/5} \vec{u},\;v = \left( {\frac{a}{{v_{f} }}} \right)Gr^{ - 1/5} \vec{v},\theta = \left( {\frac{{T - \,T_{\infty } }}{{aq_{w} /k_{f} }}} \right)Gr^{1/5} ,$$

the physical expressions ${\text{Re}} = U_{\infty } a/v_{f}$, $\Pr = \frac{{v_{f} }}{{\alpha_{f} }}$, $Q_{R} = - \frac{4\tau }{{3\gamma }}\frac{{\partial T^{4} }}{{\partial \vec{\eta }}} = \frac{16\tau }{{3\gamma }}T^{3} \frac{\partial T}{{\partial \eta }}$ are called the Reynolds number, the Prandtl number, and the Rosseland diffusion approximation for radiation, respectively, where *τ* and *γ* are Stefan–Boltzmann and mean absorption coefficients. The hybrid nanofluids and mono nanofluids thermo-physical characteristics are presented in Table 1.

**Table 1:** Thermo-physical characteristics^54^.

Characteristics of the mono nanofluid	Characteristics of the hybrid nanofluid
$\rho_{F - nf} = \left( {1 - \chi_{{\text{Fe}_{3} {\text{O}}_{4} }} } \right)\rho_{f} + \chi_{{\text{Fe}_{3} {\text{O}}_{4} }} \rho_{{\text{Fe}_{3} {\text{O}}_{4} }}$	$\rho_{F - Hnf} = \left( {1 - \chi_{{\text{Al}_{2} {\text{O}}_{3} }} } \right)\left[ {\left( {1 - \chi_{{\text{Fe}_{3} {\text{O}}_{4} }} } \right)\rho_{f} + \chi_{{\text{Fe}_{3} {\text{O}}_{4} }} \rho_{{\text{Fe}_{3} {\text{O}}_{4} }} } \right] + \chi_{{\text{Al}_{2} {\text{O}}_{3} }} \rho_{{\text{Al}_{2} {\text{O}}_{3} }}$
$$\begin{aligned} \left( {\rho c_{p} } \right)_{F - nf} = & \left( {1 - \chi_{{\text{Fe}_{3} {\text{O}}_{4} }} } \right)\left( {\rho c_{p} } \right)_{f} \\ & + \chi_{{\text{Fe}_{3} {\text{O}}_{4} }} \left( {\rho c_{p} } \right)_{{\text{Fe}_{3} {\text{O}}_{4} }} \\ \end{aligned}$$	$$\begin{aligned} \left( {\rho c_{p} } \right)_{F - Hnf} = & \left( {1 - \chi_{{\text{Al}_{2} {\text{O}}_{3} }} } \right)[\left( {1 - \chi_{{\text{Fe}_{3} {\text{O}}_{4} }} } \right)(\rho c_{p} )_{f} \\ & + \chi_{{\text{Fe}_{3} {\text{O}}_{4} }} (\rho c_{p} )_{{\text{Fe}_{3} {\text{O}}_{4} }} ] + \chi_{{\text{Al}_{2} {\text{O}}_{3} }} (\rho c_{p} )_{{\text{Al}_{2} {\text{O}}_{3} }} \\ \end{aligned}$$
$\beta_{F - nf} = \left( {1 - \chi_{{\text{Fe}_{3} {\text{O}}_{4} }} } \right)\beta_{f} + \chi_{{\text{Fe}_{3} {\text{O}}_{4} }} \beta_{{\text{Fe}_{3} {\text{O}}_{4} }}$	$\beta_{F - Hnf} = \left( {1 - \chi_{{\text{Al}_{2} {\text{O}}_{3} }} } \right)\left[ {\left( {1 - \chi_{{\text{Fe}_{3} {\text{O}}_{4} }} } \right)\beta_{f} + \chi_{{\text{Fe}_{3} {\text{O}}_{4} }} \beta_{{\text{Fe}_{3} {\text{O}}_{4} }} } \right] + \chi_{{\text{Al}_{2} {\text{O}}_{3} }} \beta_{{\text{Al}_{2} {\text{O}}_{3} }}$
$\mu_{F - nf} = \frac{{\mu_{f} }}{{\left( {1 - \chi_{{\text{Fe}_{3} {\text{O}}_{4} }} } \right)^{2.5} }}$	$\mu_{F - Hnf} = \frac{{\mu_{f} }}{{\left( {1 - \chi_{{\text{Fe}_{3} {\text{O}}_{4} }} } \right)^{2.5} \left( {1 - \chi_{{\text{Fe}_{3} {\text{O}}_{4} }} } \right)^{2.5} }}$
$\frac{{k_{F - nf} }}{{k_{f} }} = \frac{{\left( {k_{{\text{Fe}_{3} {\text{O}}_{4} }} + 2k_{f} } \right) - 2\chi_{{\text{Fe}_{3} {\text{O}}_{4} }} \left( {k_{f} - k_{{\text{Fe}_{3} {\text{O}}_{4} }} } \right)}}{{\left( {k_{{\text{Fe}_{3} {\text{O}}_{4} }} + 2k_{f} } \right) + \chi_{{\text{Fe}_{3} {\text{O}}_{4} }} \left( {k_{f} - k_{{\text{Fe}_{3} {\text{O}}_{4} }} } \right)}}$	$\,\frac{{k_{F - Hnf} }}{{k_{bf} }} = \frac{{k_{{\text{Al}_{2} {\text{O}}_{3} }} + 2k_{bf} - 2\chi_{{\text{Al}_{2} {\text{O}}_{3} }} \left( {k_{bf} - k_{{\text{Fe}_{3} {\text{O}}_{4} }} } \right)}}{{k_{{\text{Al}_{2} {\text{O}}_{3} }} + 2k_{bf} + \chi_{{\text{Al}_{2} {\text{O}}_{3} }} \left( {k_{bf} - k_{{\text{Fe}_{3} {\text{O}}_{4} }} } \right)}}$ $\,\frac{{k_{bf} }}{{k_{f} }} = \frac{{k_{{\text{Al}_{2} {\text{O}}_{3} }} + 2k_{f} - 2\chi_{{\text{Al}_{2} {\text{O}}_{3} }} \left( {k_{f} - k_{{\text{Al}_{2} {\text{O}}_{3} }} } \right)}}{{k_{{\text{Al}_{2} {\text{O}}_{3} }} + 2k_{f} + \chi_{{\text{Al}_{2} {\text{O}}_{3} }} \left( {k_{f} - k_{{\text{Al}_{2} {\text{O}}_{3} }} } \right)}}$
$\alpha_{F - nf} = \frac{{k_{F - nf} }}{{\left( {\rho c_{p} } \right)_{F - nf} }}$	$\alpha_{F - Hnf} = \frac{{k_{F - Hnf} }}{{\left( {\rho c_{p} } \right)_{F - Hnf} }}$
$$\begin{array}{*{20}l} {\frac{{\sigma_{F - nf} }}{{\sigma_{f} }} = 1 + \frac{{3(\sigma - 1)\chi_{{\text{Fe}_{3} {\text{O}}_{4} }} }}{{(\sigma + 2) - (\sigma - 1)\chi_{{\text{Fe}_{3} {\text{O}}_{4} }} }}} \hfill \\ {\sigma = \frac{{\sigma_{{\text{Fe}_{3} {\text{O}}_{4} }} }}{{\sigma_{f} }}} \hfill \\ \end{array}$$	$$\begin{gathered} \frac{{\sigma_{F - Hnf} }}{{\sigma_{bf} }} = \frac{{\sigma_{{\text{Al}_{2} {\text{O}}_{3} }} + 2\sigma_{bf} - 2\chi_{{\text{Al}_{2} {\text{O}}_{3} }} (\sigma_{bf} - \sigma_{{\text{Al}_{2} {\text{O}}_{3} }} )}}{{\sigma_{{\text{Al}_{2} {\text{O}}_{3} }} + 2\sigma_{bf} + \chi_{{\text{Al}_{2} {\text{O}}_{3} }} (\sigma_{bf} - \sigma_{{\text{Al}_{2} {\text{O}}_{3} }} )}} \hfill \\ \frac{{\sigma_{bf} }}{{\sigma_{f} }} = \frac{{\sigma_{{\text{Fe}_{3} {\text{O}}_{4} }} + 2\sigma_{f} - 2\chi_{{\text{Fe}_{3} {\text{O}}_{4} }} (\sigma_{f} - \sigma_{{\text{Fe}_{3} {\text{O}}_{4} }} )}}{{\sigma_{{\text{Fe}_{3} {\text{O}}_{4} }} + 2\sigma_{f} + \chi_{{\text{Fe}_{3} {\text{O}}_{4} }} (\sigma_{f} - \sigma_{{\text{Fe}_{3} {\text{O}}_{4} }} )}} \hfill \\ \end{gathered}$$

As in works of literature^55–57^, where they handled hybrid nanofluid characteristics, in Table 1, with the aid of Eq. (12), the Eqs. (8)–(10) that depict radiation impacts on electro-conductive Williamson hybrid ferronanofluid under the magnetic field can be rewritten as:

13 $$\frac{\partial u}{{\partial \omega }} + \frac{\partial v}{{\partial \eta }} = 0,$$

14 $$\begin{aligned} u\frac{\partial u}{{\partial \omega }} + v\frac{\partial u}{\eta } = & \frac{{\rho_{f} }}{{\rho_{F - Hnf} }}\left( {\frac{1}{{(1 - \chi_{{\text{Al}_{2} {\text{O}}_{3} }} )^{2.5} (1 - \chi_{{\text{Fe}_{3} {\text{O}}_{4} }} )^{2.5} }}} \right)\frac{{\partial^{2} u}}{{\partial \eta^{2} }} + {\text{We}}\left( {\frac{{\partial^{2} u}}{{\partial \omega^{2} }}\frac{\partial u}{{\partial \omega }}} \right) \\ & + \frac{1}{{\rho_{F - Hnf} }}\left( {\left( {1 - \chi_{{\text{Al}_{2} {\text{O}}_{3} }} } \right)\left[ {\left( {1 - \chi_{{\text{Fe}_{3} {\text{O}}_{4} }} } \right)\rho_{f} + \chi_{{\text{Al}_{2} {\text{O}}_{3} }} \frac{{\rho_{{\text{Al}_{2} {\text{O}}_{3} }} \beta_{{\text{Al}_{2} {\text{O}}_{3} }} }}{{\beta_{f} }}} \right] + \chi_{{\text{Fe}_{3} {\text{O}}_{4} }} \frac{{\rho_{{\text{Fe}_{3} {\text{O}}_{4} }} \beta_{{\text{Fe}_{3} {\text{O}}_{4} }} }}{{\beta_{f} }}} \right) \\ & \times \lambda \theta \sin \omega - \,\frac{{\rho_{f} }}{{\rho_{F - Hnf} }}\,\frac{{\sigma_{F - Hnf} }}{{\sigma_{f} }}{\text{M}}u, \\ \end{aligned}$$

15 $$\left( {\frac{\Pr }{{1 + (3/4){\text{R}}}}} \right)\left( {u\frac{\partial \theta }{{\partial \omega }} + v\frac{\partial \theta }{{\partial \eta }}} \right) = \left[ {\frac{{{{k_{F - Hnf} } \mathord{\left/ {\vphantom {{k_{F - Hnf} } {k_{f} }}} \right. \kern-0pt} {k_{f} }}}}{{\left( {1 - \chi_{{\text{Al}_{2} {\text{O}}_{3} }} } \right)[\left( {1 - \chi_{{\text{Fe}_{3} {\text{O}}_{4} }} } \right) + \chi_{{\text{Fe}_{3} {\text{O}}_{4} }} \frac{{(\rho Cp)_{{\text{Fe}_{3} {\text{O}}_{4} }} }}{{(\rho Cp)_{f} }}] + \chi_{{\text{Al}_{2} {\text{O}}_{3} }} \frac{{(\rho Cp)_{{\text{Al}_{2} {\text{O}}_{3} }} }}{{(\rho Cp)_{f} }}}}} \right]\frac{{\partial^{2} \theta }}{{\partial n^{2} }}.$$

The boundary condition (11) is generated in the sense:

16 $$\begin{array}{*{20}l} {u = v = 0,\,\,\theta ^{\prime} = - 1,\,\,{\text{at}}\,\,\eta = 0,} \hfill \\ {u \to 0,\,\,\theta \to 0,\,\,{\text{as}}\,\,\eta \to \infty .} \hfill \\ \end{array}$$

In the above equations, the Weissenberg number ${\text{We}} = \frac{{\sqrt 2 \Gamma v\eta \,Gr^{3/5} }}{{a^{3} }}$, and the magnetic parameter ${\text{M}} = \left( {\frac{{\sigma_{f} B_{o}^{2} a^{2} {\text{Gr}}^{ - 2/5} }}{{\rho_{f} v_{f} }}} \right)$.

By using the following similarity transformations, which are determined as:

$$\psi = \omega f(\omega ,\eta ),\,\,\,\theta = \theta (\omega ,\eta ),\,$$

17 $$u = \frac{\partial \psi }{{\partial \omega }}\;{\text{and}}\;v = - \frac{\partial \psi }{{\partial \eta }},$$

where $\psi$ is the stream function.

By taking advantage of the previous transformation variables (17), the dimensionless governing Eqs. (12) and (13) are converted into the following PDEs:

18 $$\begin{aligned} & \frac{{\rho_{f} }}{{\rho_{F - Hnf} }}\left( {\frac{1}{{(1 - \chi_{{(Al_{2} {\text{O}}_{3} }} )^{2.5} (1 - \chi_{{\text{Fe}_{3} {\text{O}}_{4} }} )^{2.5} }}} \right)\frac{{\partial^{3} f}}{{\partial \eta^{3} }} + {\text{We}}\frac{{\partial^{3} f}}{{\partial \eta^{3} }}\frac{{\partial^{2} f}}{{\partial \eta^{2} }} + f\frac{{\partial^{2} f}}{{\partial \eta^{2} }} - \left( {\frac{\partial f}{{\partial \eta }}} \right)^{2} \\ &\quad+ \frac{1}{{\rho_{F - Hnf} }}\left( {\left( {1 - \chi_{{\text{Al}_{2} {\text{O}}_{3} }} } \right)\left[\left( {1 - \chi_{{\text{Fe}_{3} {\text{O}}_{4} }} } \right)\rho_{f} + \chi_{{\text{Fe}_{3} {\text{O}}_{4} }} \frac{{\rho_{{\text{Fe}_{3} {\text{O}}_{4} }} \beta_{{\text{Fe}_{3} {\text{O}}_{4} }} }}{{\beta_{f} }}\right] + \chi_{{\text{Al}_{2} {\text{O}}_{3} }} \frac{{\rho_{{\text{Al}_{2} {\text{O}}_{3} }} \beta_{{\text{Al}_{2} {\text{O}}_{3} }} }}{{\beta_{f} }}} \right)\frac{\sin \omega }{\omega }\,\theta \end{aligned}$$

19 $$\begin{aligned} & \left[ {\frac{{{{k_{F - Hnf} } \mathord{\left/ {\vphantom {{k_{F - Hnf} } {k_{f} }}} \right. \kern-0pt} {k_{f} }}}}{{\left( {1 - \chi_{{\text{Al}_{2} {\text{O}}_{3} }} } \right)\left[\left( {1 - \chi_{{\text{Fe}_{3} {\text{O}}_{4} }} } \right) + \chi_{{\text{Fe}_{3} {\text{O}}_{4} }} (\rho Cp)_{{\text{Fe}_{3} {\text{O}}_{4} }} /(\rho Cp)_{f} \right] + \chi_{{\text{Al}_{2} {\text{O}}_{3} }} (\rho Cp)_{{\text{Al}_{2} {\text{O}}_{3} }} /(\rho Cp)_{f} }}} \right]\frac{{\partial^{2} \theta }}{{\partial \eta^{2} }} \\ & \quad + \left( {\frac{\Pr }{{1 + (3/4){\text{R}}}}} \right)f\frac{\partial \theta }{{\partial \eta }} = m\left( {\frac{\partial f}{{\partial \eta }}\frac{\partial \theta }{{\partial \omega }} - \frac{\partial f}{{\partial \omega }}\frac{\partial \theta }{{\partial \eta }}} \right), \end{aligned}$$

subject to:

20 $$\begin{array}{*{20}l} {f = \frac{\partial f}{{\partial n}} = 0,\;\theta ^{\prime} = - 1\,\,{\text{at}}\,\,n = 0,} \hfill \\ {\frac{\partial f}{{\partial n}} \to 0,\,\,\theta \to 0,\,\,{\text{as}}\,\,n \to \infty .} \hfill \\ \end{array}$$

If we consider that *ω* is approximately (equal to zero stagnation point), the system of PDEs (18)–(20) converts into:

21 $$\begin{aligned} & \frac{{\rho_{f} }}{{\rho_{F - Hnf} }}\left( {\frac{1}{{(1 - \chi_{{\text{Al}_{2} {\text{O}}_{3} }} )^{2.5} (1 - \chi_{{\text{Fe}_{3} {\text{O}}_{4} }} )^{2.5} }}} \right)\frac{{\partial^{3} f}}{{\partial \eta^{3} }} + {\text{We}}\frac{{\partial^{3} f}}{{\partial \eta^{3} }}\frac{{\partial^{2} f}}{{\partial \eta^{2} }} + f\frac{{\partial^{2} f}}{{\partial \eta^{2} }} - \left( {\frac{\partial f}{{\partial \eta }}} \right)^{2} \\ &\quad + \frac{1}{{\rho_{F - Hnf} }}\left( {\left( {1 - \chi_{{\text{Al}_{2} {\text{O}}_{3} }} } \right)\left[ {\left( {1 - \chi_{{\text{Fe}_{3} {\text{O}}_{4} }} } \right)\rho_{f} + \chi_{{\text{Fe}_{3} {\text{O}}_{4} }} \frac{{\rho_{{\text{Fe}_{3} {\text{O}}_{4} }} \beta_{{\text{Fe}_{3} {\text{O}}_{4} }} }}{{\beta_{f} }}} \right] + \chi_{{\text{Al}_{2} {\text{O}}_{3} }} \frac{{\rho_{{\text{Al}_{2} {\text{O}}_{3} }} \beta_{{\text{Al}_{2} {\text{O}}_{3} }} }}{{\beta_{f} }}.} \right)\theta \\ &\quad - \frac{{\rho_{f} }}{{\rho_{F - Hnf} }}\,\frac{{\sigma_{F - Hnf} }}{{\sigma_{f} }}{\text{M}}\,\,\frac{\partial f}{{\partial \eta }} = 0, \end{aligned}$$

22 $$\frac{1}{\Pr }\left[ {\frac{{{{k_{F - Hnf} } \mathord{\left/ {\vphantom {{k_{F - Hnf} } {k_{f} }}} \right. \kern-0pt} {k_{f} }}}}{{\left( {1 - \chi_{{\text{Al}_{2} {\text{O}}_{3} }} } \right)\left[ {\left( {1 - \chi_{{\text{Fe}_{3} {\text{O}}_{4} }} } \right) + \chi_{{\text{Fe}_{3} {\text{O}}_{4} }} \frac{{(\rho Cp)_{{\text{Fe}_{3} {\text{O}}_{4} }} }}{{(\rho Cp)_{f} }}} \right] + \chi_{{\text{Al}_{2} {\text{O}}_{3} }} \frac{{(\rho Cp)_{{\text{Al}_{2} {\text{O}}_{3} }} }}{{(\rho Cp)_{f} }}}}} \right]\frac{{\partial^{2} \theta }}{{\partial \eta^{2} }} + \left( {\frac{\Pr }{{1 + (3/4){\text{R}}}}} \right)f\frac{\partial \theta }{{\partial \eta }} = 0,$$

Boundary conditions become:

23 $$\begin{array}{*{20}l} {f\left( {0,n} \right) = f^{\prime}\left( {0,n} \right) = 0,\,\theta ^{\prime}\left( {0,n} \right) = - 1\,\,{\text{as}}\,\,n = 0,} \hfill \\ {f^{\prime}\left( {0,n} \right) \to 0,\,\,\theta \left( {0,n} \right) \to 0\,\,{\text{as}}\,n \to \infty .} \hfill \\ \end{array}$$

The significant investigations of engineering quantities (the local skin friction coefficient *C*_*f*_, the local Nusselt number *Nu*) as evidenced in the previous studies^55,58^ are obtained in this consideration according to the following:

24 $$C_{f} \, = \,\left( {\frac{{\tau_{w} }}{{\rho_{f} U_{\infty }^{2} }}} \right),\;Nu = \left( {\frac{{aq_{w} }}{{k_{f} (T_{w} - T_{\infty } )}} + Q_{R} } \right),$$

where

25 $$\tau_{w} = \mu_{F - Hnf} \left( {\frac{{\partial \vec{u}}}{{\partial \vec{\eta }}} + \left[ {\frac{\Gamma }{\sqrt 2 }\left( {\frac{{\partial \vec{u}}}{{\partial \vec{\eta }}}} \right)^{2} } \right]} \right)_{{\vec{\eta } = \,0}} ,\;q_{w} = - \,k_{F - Hnf} \left( {\frac{\partial T}{{\partial \vec{\eta }}}} \right)_{{\vec{\eta } = \,0}} .$$

Similarly, employing Eqs. (24) and (25), hence *C*_*f*_ and *Nu* are attained as:

$$C_{f} = {\text{Gr}}^{ - 1/5} \frac{1\,}{{(1 - \chi_{{\text{Al}_{2} {\text{O}}_{3} }} )^{2.5} (1 - \chi_{{\text{Fe}_{3} {\text{O}}_{4} }} )^{2.5} }}\omega \left( {\frac{{\partial^{2} f}}{{\partial \eta^{2} }}\,(\omega ,0) + \frac{{{\text{We}}}}{2}\left( {\frac{\partial f}{{\partial \eta }}(\omega ,0)} \right)^{2} } \right),$$

26 $$Nu = \,{\text{Gr}}^{1/5} \left( {1 + \frac{4}{3}R} \right)\frac{{k_{F - Hnf} }}{{k_{f} }}\left( {\frac{1}{\theta (\omega ,0)}} \right).$$

The studied parameters and symbols are displayed in the nomenclature list.

## Numerical method

In the previous section, dimensional PDEs with constant heat flow boundary conditions were converted by suitable variables into nondimensional PDEs. Consequently, these PDEs need an efficient and accurate numerical method. The Keller box method was relied upon in order to find an approximate numerical solution to these equations^59–61^. Firstly, it can be written the equations by reducing them to a first-order system using the finite difference scheme, as follows:

27 $$\begin{array}{*{20}l} u\left( {\omega ,\eta } \right) = f^{\prime}\left( {\omega ,\eta } \right) \to (u = f^{\prime}), \hfill \\ v\left( {\omega ,\eta } \right) = f^{\prime\prime}\left( {\omega ,\eta } \right) \to (v = f^{\prime\prime} = u^{\prime}), \hfill \\ t^{\prime}(\omega ,\eta ) = \theta (\omega ,\eta ) \to t = \theta ^{\prime}, \hfill \\ \end{array}$$

The above partial differential Eqs. (18) and (19) to be displayed are:

28 $$\begin{aligned} & \frac{{\rho_{f} }}{{\rho_{F - Hnf} }}\left( {\frac{1}{{(1 - \chi_{{\text{Al}_{2} {\text{O}}_{3} }} )^{2.5} (1 - \chi_{{\text{Fe}_{3} {\text{O}}_{4} }} )^{2.5} }}} \right)v^{\prime} + {\text{We}}\,\,v^{\prime}v + f\,v - \left( u \right)^{2} \\ &\quad+ \frac{1}{{\rho_{F - Hnf} }}\left( {\left( {1 - \chi_{{\text{Al}_{2} {\text{O}}_{3} }} } \right)\left[ {\left( {1 - \chi_{{\text{Fe}_{3} {\text{O}}_{4} }} } \right)\rho_{f} + \chi_{{\text{Fe}_{3} {\text{O}}_{4} }} \frac{{\rho_{{\text{Fe}_{3} {\text{O}}_{4} }} \beta_{{\text{Fe}_{3} {\text{O}}_{4} }} }}{{\beta_{f} }}} \right] + \chi_{{\text{Al}_{2} {\text{O}}_{3} }} \frac{{\rho_{{\text{Al}_{2} {\text{O}}_{3} }} \beta_{{\text{Al}_{2} {\text{O}}_{3} }} }}{{\beta_{f} }}} \right)\theta \\ &\quad- \frac{{\rho_{f} }}{{\rho_{F - Hnf} }}\,\frac{{\sigma_{F - Hnf} }}{{\sigma_{f} }}{\text{M}}\,\,u\, = \omega \left( {u\frac{\partial u}{{\partial \omega }} - \frac{\partial f}{{\partial \omega }}v} \right), \end{aligned}$$

29 $$\frac{1}{\Pr }\left[ {\frac{{{{k_{F - Hnf} } \mathord{\left/ {\vphantom {{k_{F - Hnf} } {k_{f} }}} \right. \kern-0pt} {k_{f} }}}}{{\left( {1 - \chi_{{\text{Al}_{2} {\text{O}}_{3} }} } \right)[\left( {1 - \chi_{{\text{Fe}_{3} {\text{O}}_{4} }} } \right) + \chi_{{\text{Fe}_{3} {\text{O}}_{4} }} \frac{{(\rho Cp)_{{\text{Fe}_{3} {\text{O}}_{4} }} }}{{(\rho Cp)_{f} }}] + \chi_{{\text{Al}_{2} {\text{O}}_{3} }} \frac{{(\rho Cp)_{{\text{Al}_{2} {\text{O}}_{3} }} }}{{(\rho Cp)_{f} }}}}} \right]\theta ^{\prime\prime} + \left( {\frac{\Pr }{{1 + (3/4){\text{R}}}}} \right)f\frac{\partial \theta }{{\partial \eta }} = \omega \left( {f^{\prime}\frac{\partial \theta }{{\partial \omega }} - \frac{\partial f}{{\partial \omega }}\theta ^{\prime}} \right).$$

The primes symbol signalizes the partial derivative of *η*. Also, the boundary conditions (20) are presented as follows:

30 $$\begin{array}{*{20}l} {f\left( {\omega ,0} \right) = 0,f^{\prime}\left( {\omega ,0} \right) = 0,\,\theta ^{\prime} = - 1,} \hfill \\ {f^{\prime}\left( {\omega ,\infty } \right) = 0,\theta \left( {\omega ,\infty } \right) = 0.} \hfill \\ \end{array}$$

**Figure 2:**
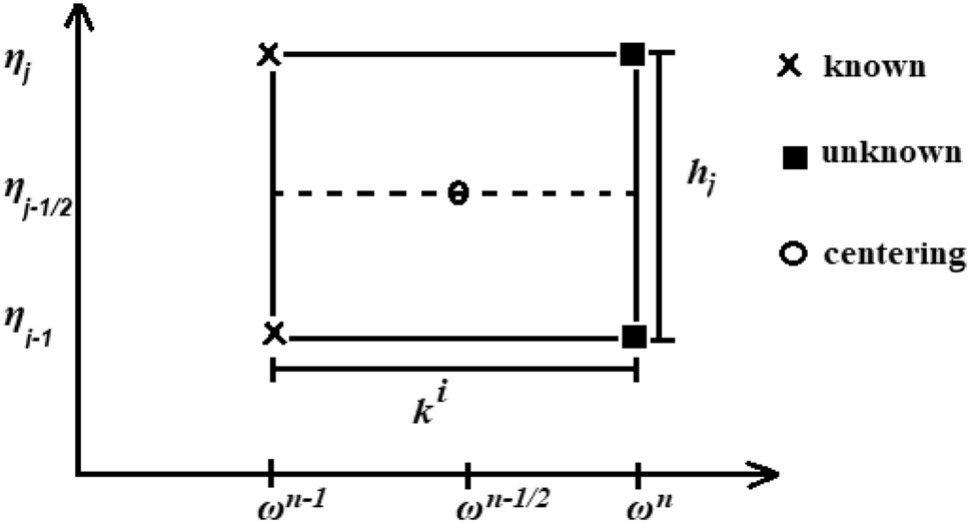
Net rectangle for difference approximations.

To understand the mesh points procedures in a two-dimensional *ω-η* plane, suppose the step sizes (*k*^*n*^ and *h*_*n*_) of the concerned two-dimensional ω and η directions, respectively, as offered in Fig. 2.

The mesh points can be summarized as:

31 $$\begin{array}{*{20}l} \omega^{0} = 0,\,\,\omega^{i} = \omega^{i} = \omega^{i - 1} + k^{i} ,i = 0,1,2,3,...,N, \hfill \\ \eta_{0} = 0,\,\,\eta_{j} = \eta_{j - 1} + h_{j} ,j = 0,1,2,3,...,J.\eta_{\infty } = \eta_{J} . \hfill \\ \end{array}$$

We also utilize the notation ( ) for the points and independent quantities midpoint and first derivative in the *ω*-orientation and *η*-orientation, introduced by finite difference, as follows:

32 $$\begin{array}{*{20}l} \left( {} \right)_{{j - {1 \mathord{\left/ {\vphantom {1 2}} \right. \kern-0pt} 2}}}^{{n - {1 \mathord{\left/ {\vphantom {1 2}} \right. \kern-0pt} 2}}} = \frac{1}{4}\left( {\left( {} \right)_{j}^{n} + \left( {} \right)_{j - 1}^{n} + \left( {} \right)_{j}^{n - 1} + \left( {} \right)_{j - 1}^{n - 1} } \right), \hfill \\ \left( {\frac{{\partial \left( {} \right)}}{\partial \eta }} \right)_{{j - {1 \mathord{\left/ {\vphantom {1 2}} \right. \kern-0pt} 2}}}^{{n - {1 \mathord{\left/ {\vphantom {1 2}} \right. \kern-0pt} 2}}} = \frac{1}{{2h_{j} }}\left( {\left( {} \right)_{j}^{n} - \left( {} \right)_{j - 1}^{n} + \left( {} \right)_{j}^{n - 1} - \left( {} \right)_{j - 1}^{n - 1} } \right), \hfill \\ \left( {\frac{{\partial \left( {} \right)}}{\partial \omega }} \right)_{{j - {1 \mathord{\left/ {\vphantom {1 2}} \right. \kern-0pt} 2}}}^{{n - {1 \mathord{\left/ {\vphantom {1 2}} \right. \kern-0pt} 2}}} = \frac{1}{{2k^{n} }}\left( {\left( {} \right)_{j}^{n} - \left( {} \right)_{j - 1}^{n} + \left( {} \right)_{j}^{n - 1} - \left( {} \right)_{j - 1}^{n - 1} } \right). \hfill \\ \end{array}$$

The resulted finite difference approximations for Eqs. (27)–(29) can be extracted at the midpoint (*ωn, ηj−*1/2), which is named “centering”. And then, we obtain

33 $$f_{j}^{n} - f_{j - 1}^{n} = h_{j} \left( {u_{j - 1/2}^{n} } \right),$$

34 $$u_{j}^{n} - u_{{j - 1}}^{n} = h_{j} \left( {v_{{j - 1/2}}^{n} } \right),$$

35 $$\theta_{j}^{n} - \theta_{j - 1}^{n} = h_{j} \left( {t_{j - 1/2}^{n} } \right),$$

36 $$\begin{aligned} & \frac{{\rho_{f} }}{{\rho_{F - Hnf} }}\left( {\frac{1}{{(1 - \chi_{{\text{Al}_{2} {\text{O}}_{3} }} )^{2.5} (1 - \chi_{{\text{Fe}_{3} {\text{O}}_{4} }} )^{2.5} }}} \right)\,\left( {v_{j}^{{}} - v_{j - 1}^{{}} } \right) + {\text{We}}\left( {v_{j}^{{}} + v_{j - 1}^{{}} } \right)\left( {v_{j}^{{}} - v_{j - 1}^{{}} } \right) \\ &\quad + \frac{1 + \varepsilon }{4}h_{j} (f_{j}^{{}} + f_{j - 1}^{{}} )(v_{j}^{{}} + v_{j - 1}^{{}} ) - \,\left( {\frac{1 + \varepsilon }{4}} \right)h_{j} \,(u_{j}^{{}} + u_{j - 1}^{{}} )^{2} - \frac{1}{2}\frac{{\rho_{f} }}{{\rho_{F - Hnf} }}\,\frac{{\sigma_{F - Hnf} }}{{\sigma_{f} }}\,{\text{M}}h_{j} \,\,(u_{j}^{{}} + u_{j - 1}^{{}} ) \\ & \quad + \,\left( {\frac{\varepsilon }{2}} \right)h_{j} v_{j - 1/2}^{n - 1} (f_{j}^{{}} + f_{j - 1}^{{}} )\, - \left( {\frac{\varepsilon }{2}} \right)h_{j} f_{j - 1/2}^{n - 1} (v_{j}^{{}} + v_{j - 1}^{{}} )f_{j - 1/2}^{n - 1} + \,\frac{1}{2}\frac{1}{{\rho_{F - Hnf} }}\left( \begin{gathered} \left( {1 - \chi_{{\text{Al}_{2} {\text{O}}_{3} }} } \right)[\left( {1 - \chi_{{\text{Fe}_{3} {\text{O}}_{4} }} } \right)\rho_{f} \hfill \\ + \chi_{{\text{Fe}_{3} {\text{O}}_{4} }} \frac{{\rho_{{\text{Fe}_{3} {\text{O}}_{4} }} \beta_{{\text{Fe}_{3} {\text{O}}_{4} }} }}{{\beta_{f} }}] \hfill \\ + \chi_{{\text{Al}_{2} {\text{O}}_{3} }} \frac{{\rho_{{\text{Al}_{2} {\text{O}}_{3} }} \beta_{{\text{Al}_{2} {\text{O}}_{3} }} }}{{\beta_{f} }} \hfill \\ \end{gathered} \right)\\ &\quad\frac{{\sin \omega^{n - 1/2} }}{{\omega^{n - 1/2} }}h_{j} (\theta_{j} + \theta_{j - 1} )\, = \left( {L_{1} } \right)_{j - 1/2}^{n - 1} , \end{aligned}$$

37 $$\begin{aligned} & \frac{1}{\Pr }\left[ {\frac{{{{k_{F - Hnf} } \mathord{\left/ {\vphantom {{k_{F - Hnf} } {k_{f} }}} \right. \kern-0pt} {k_{f} }}}}{{\left( {1 - \chi_{{\text{Al}_{2} {\text{O}}_{3} }} } \right)[\left( {1 - \chi_{{\text{Fe}_{3} {\text{O}}_{4} }} } \right) + \chi_{{\text{Fe}_{3} {\text{O}}_{4} }} \frac{{(\rho Cp)_{{\text{Fe}_{3} {\text{O}}_{4} }} }}{{(\rho Cp)_{f} }}] + \chi_{{\text{Al}_{2} {\text{O}}_{3} }} \frac{{(\rho Cp)_{{\text{Al}_{2} {\text{O}}_{3} }} }}{{(\rho Cp)_{f} }}}}} \right]\left( {t_{j}^{{}} - t_{j - 1}^{{}} } \right)\, \\ &\quad- \frac{\varepsilon }{4}h_{j} (u_{j}^{{}} + u_{j - 1}^{{}} )(\theta_{j}^{{}} + \theta_{j - 1}^{{}} ) + \frac{1 + \varepsilon }{4}h_{j} (f_{j}^{{}} + f_{j - 1}^{{}} )(t_{j}^{{}} + t_{j - 1}^{{}} ) + \frac{\varepsilon }{2}h_{j} (u_{j}^{{}} + u_{j - 1}^{{}} )\theta_{j - 1/2}^{n - 1} \\ &\quad- \frac{\varepsilon }{2}h_{j} u_{j - 1/2}^{n - 1} (\theta_{j}^{{}} + \theta_{j - 1}^{{}} ) - \frac{\varepsilon }{2}h_{j} (t_{j}^{{}} - t_{j - 1}^{{}} )f_{j - 1/2}^{n - 1} + \frac{\varepsilon }{2}\left( {\frac{\Pr }{{1 + (3/4){\text{R}}}}} \right)h_{j} t_{j - 1/2}^{n - 1} (f_{j}^{{}} + f_{j - 1}^{{}} ) = \left( {L_{2} } \right)_{j - 1/2}^{n - 1} , \end{aligned}$$

38 $$\begin{aligned} \left( {L_{1} } \right)_{j - 1/2}^{n - 1} \, &= - h_{j} \left(\vphantom{\left( \begin{gathered} \left( {1 - \chi_{{\text{Al}_{2} {\text{O}}_{3} }} } \right)[\left( {1 - \chi_{{\text{Fe}_{3} {\text{O}}_{4} }} } \right)\rho_{f} + \hfill \\ \chi_{{\text{Fe}_{3} {\text{O}}_{4} }} \frac{{\rho_{{\text{Fe}_{3} {\text{O}}_{4} }} \beta_{{\text{Fe}_{3} {\text{O}}_{4} }} }}{{\beta_{f} }}] \hfill \\ + \chi_{{\text{Al}_{2} {\text{O}}_{3} }} \frac{{\rho_{{\text{Al}_{2} {\text{O}}_{3} }} \beta_{{\text{Al}_{2} {\text{O}}_{3} }} }}{{\beta_{f} }} \hfill \\ \end{gathered} \right)} {\frac{{\rho_{f} }}{{\rho_{F - Hnf} }}\left( {\frac{1}{{(1 - \chi_{{\text{Al}_{2} {\text{O}}_{3} }} )^{2.5} (1 - \chi_{{\text{Fe}_{3} {\text{O}}_{4} }} )^{2.5} }}} \right)\,\,\frac{{\left( {v_{j}^{{}} - v_{j - 1}^{{}} } \right)}}{{h_{j} }} + {\text{We}}\,v_{j - 1} v^{\prime}_{j - 1/2} }\right. \\&\quad \left.{+ (1 - \varepsilon )f_{j - 1/2}^{{}} v_{j - 1/2}^{{}}+ (\varepsilon - \,1)\left( {\,u_{j - 1/2}^{{}} } \right)^{2} - \frac{{\rho_{f} }}{{\rho_{F - Hnf} }}\,\frac{{\sigma_{F - Hnf} }}{{\sigma_{f} }}\,{\text{M}}u_{j - 1/2}^{{}} \, }\right. \\&\quad \left.{ + \frac{1}{{\rho_{F - Hnf} }}\left( \begin{gathered} \left( {1 - \chi_{{\text{Al}_{2} {\text{O}}_{3} }} } \right)[\left( {1 - \chi_{{\text{Fe}_{3} {\text{O}}_{4} }} } \right)\rho_{f} + \hfill \\ \chi_{{\text{Fe}_{3} {\text{O}}_{4} }} \frac{{\rho_{{\text{Fe}_{3} {\text{O}}_{4} }} \beta_{{\text{Fe}_{3} {\text{O}}_{4} }} }}{{\beta_{f} }}] \hfill \\ + \chi_{{\text{Al}_{2} {\text{O}}_{3} }} \frac{{\rho_{{\text{Al}_{2} {\text{O}}_{3} }} \beta_{{\text{Al}_{2} {\text{O}}_{3} }} }}{{\beta_{f} }} \hfill \\ \end{gathered} \right)\frac{{\sin \omega^{n - 1/2} }}{{\omega^{n - 1/2} }}\theta_{j - 1/2} } \right)^{n - 1} , \end{aligned}$$

39 $$\begin{aligned} \left( {L_{2} } \right)_{j}^{n - 1} & = \, - h_{j} \left( {\frac{1}{\Pr }\left[ {\frac{{k_{F - Hnf} /k_{f} }}{{\left( {1 - \chi_{{\text{Al}_{2} {\text{O}}_{3} }} } \right)\left[ {\left( {1 - \chi_{{\text{Fe}_{3} {\text{O}}_{4} }} } \right) + \chi_{{\text{Fe}_{3} {\text{O}}_{4} }} \frac{{(\rho Cp)_{{\text{Fe}_{3} {\text{O}}_{4} }} }}{{(\rho Cp)_{f} }}} \right] + \chi_{{\text{Al}_{2} {\text{O}}_{3} }} \frac{{(\rho Cp)_{{\text{Al}_{2} {\text{O}}_{3} }} }}{{(\rho Cp)_{f} }}}}} \right]\frac{{\left( {t_{j} - t_{j - 1} } \right)}}{{h_{j} }} }\right.\\ & \quad \left.{+ \left( {1 - \varepsilon } \right)f_{j - 1/2} t_{j - 1/2} + \varepsilon u_{j - 1/2} \theta_{j - 1/2} } \vphantom{{\frac{1}{\Pr }\left[ {\frac{{k_{F - Hnf} /k_{f} }}{{\left( {1 - \chi_{{\text{Al}_{2} {\text{O}}_{3} }} } \right)\left[ {\left( {1 - \chi_{{\text{Fe}_{3} {\text{O}}_{4} }} } \right) + \chi_{{\text{Fe}_{3} {\text{O}}_{4} }} \frac{{(\rho Cp)_{{\text{Fe}_{3} {\text{O}}_{4} }} }}{{(\rho Cp)_{f} }}} \right] + \chi_{{\text{Al}_{2} {\text{O}}_{3} }} \frac{{(\rho Cp)_{{\text{Al}_{2} {\text{O}}_{3} }} }}{{(\rho Cp)_{f} }}}}} \right]\frac{{\left( {t_{j} - t_{j - 1} } \right)}}{{h_{j} }} }}\right)^{n - 1} , \end{aligned}$$

where $$\varepsilon = \frac{{\omega^{n - 1/2} }}{{k_{n} }}.$$

The boundary condition can be rewritten as:

40 $$f_{0}^{n} \, = \,u_{0}^{n} = 0,\,t_{0}^{n} = 1,u_{J}^{n} = \theta_{J}^{n} = 0.$$

Consequently, the gained finite difference equations will be transformed into the linear system of equations, via Newton’s method, and rearranged them in a matrix–vector form. Finally, the block tri-diagonal elimination technique will be used to solve the matrix–vector form to achieve the most elaborate approximation numerical results. These numerical results will be shown as figures and tables by the Matlab program. Once the MATLAB program code has been established, it is required to identify the convergence criteria, which requires us to recognize some specific computations: the appropriate steps size ∆*η* and ∆*ω*, as well as the boundary layer thickness *ω*_∞_, In the current study *ω*_∞_ properly sets between 1.3 and 6, to get the boundary layer convergence. Once the suitable value of *ω*_∞_ is fixed, we determine the step size ∆*η* = 0.005 and step size ∆*ω* = 0.02, which are convenient to gain accurate approximate numerical outcomes. Further, these values are utilized to get numerical results almost compatible with previous literature, as shown in Tables 2 and 3. On the other hand, the computations are iterated until convergence criteria are satisfied. The efficient component *v*(*η*,0), is generally applied as the convergence criterion in laminar boundary layer computations (see^57^). The computations are terminated when |∆*v*_*o*_(*η*,0)|< 10^−5^.

**Table 2:** Comparison of the present outcomes of the local skin friction values *C*_*f*_ with previous published literature.

$\eta$	Merkin and Po^52^	Current findings	$\xi$
0	0.000	0.000	0.0000
0.2	0.274	0.274	0.0000
0.6	0.795	0.793	0.2522
1.0	1.241	1.241	0.0000
1.6	1.671	1.668	0.1799
2.0	1.744	1.716	1.6317
2.6	1.451	1.410	2.9078
3.0	0.913	0.897	0.1783
$\pi$	0.613	0.600	0.2167

**Table 3:** Comparison of the present outcomes of the local Nusselt number *Nu* with previous published literature.

$\eta$	Merkin and Pop^52^	Current findings	$\xi$
0	1.996	1.996	0.0000
0.2	1.999	1.999	0.0000
0.6	2.014	2.015	0.0496
1.0	2.043	2.046	0.1466
1.6	2.120	2.129	0.4227
2.0	2.202	2.215	0.5869
2.6	2.403	2.420	0.7025
3.0	2.660	2.689	1.0108
Π	2.824	2.837	0.4582

## Results and discussion

This section discusses and analyzes the impressions and tendencies of physical quantities associated with free convection, as well as their responses when the magnetic field strength ${\text{M,}}$ thermal radiation factor ${\text{R,}}$ volume fraction of ultrafine particle $\chi ,$ and Weissenberg number ${\text{We}}$ are increased. The approximate values of skin friction *C*_*f*_ and Nusselt number *Nu*, were compared with prior published findings in the literature at fixed parameters. In addition, the following formula is used to calculate the approximate relative error $\xi$ between the current findings ($r_{c}$) and previous findings (Merkin and Pop52 ($r_{p}$)), are found using the formula:

$$\xi = \frac{{\left| {r_{c} - \left. {r_{p} } \right|} \right.}}{{r_{c} }} \times 100\% .$$

See Tables 2 and 3.

Our results achieved an excellent compatible with those previously published. Table 4 shows the thermo-physical features of engine oil and used nanoparticles.

**Table 4:** Thermo-physical features of engine oil and used nanoparticles^18,57,62,63^.

Thermo-physical feature	Engine oil	$Al_{2} {\text{O}}_{3}$	$Fe_{3} {\text{O}}_{4}$
*C*_*p*_ (J/kg K)	1910	765	670
*β* × 10^−5^ (K^−1^)	70	0.85	20.6
*ρ* (kg/m^3^)	884	3970	5180
*K* (W/m K)	0.114	40	80.4
*σ* (s/m)	1.07 × 10^–6^	3.5 × 10^7^	1.12 × 10^5^
Pr	6450	–	

**Figure 3:**
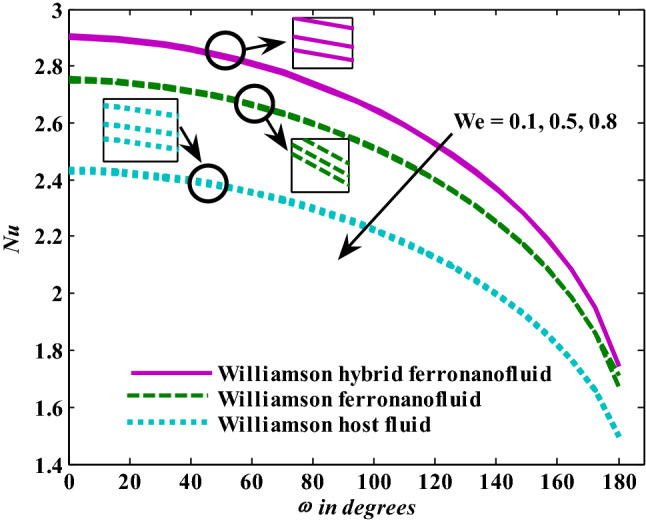
Nusselt number responses as a result of ${\text{We}}$ increasing at fixed ${\text{R}} = 3,\,{\text{M}} = 0.8,\,\chi_{{\text{Fe}_{3} {\text{O}}_{4} }} = 0.1,\,\chi_{{\text{Al}_{2} {\text{O}}_{3} }} = 0.05$.

**Figure 4:**
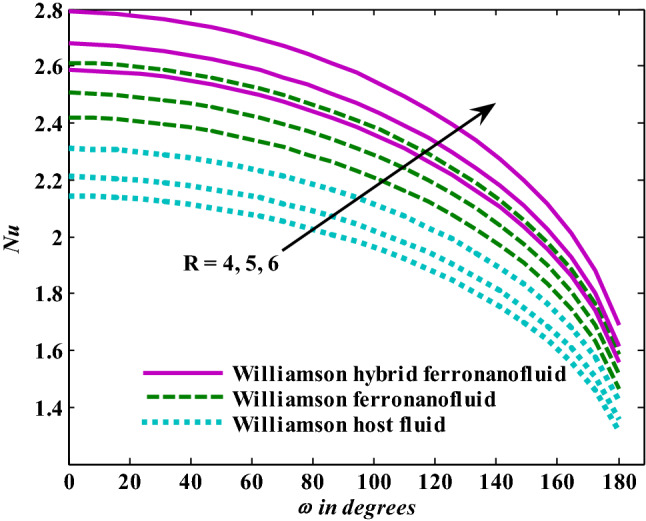
Nusselt number responses as a result of ${\text{R}}$ increasing at fixed ${\text{We}} = 0.{7},{\text{ M = }}0.{8},\chi_{{\text{Fe}_{3} {\text{O}}_{4} }} = 0.1,\,\chi_{{\text{Al}_{2} {\text{O}}_{3} }} = 0.05$.

**Figure 5:**
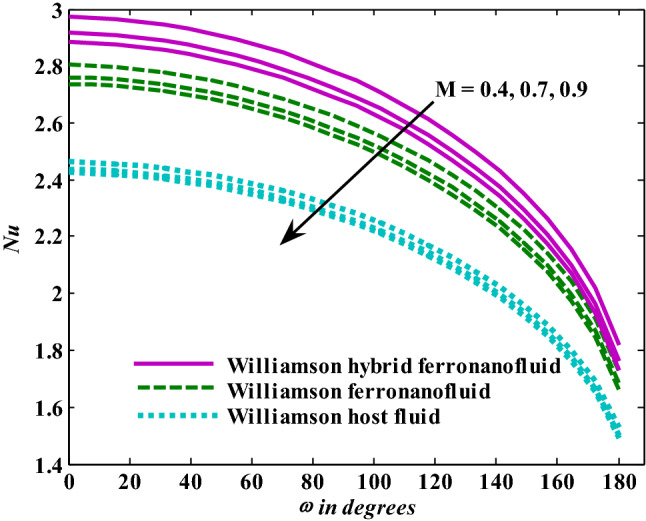
Nusselt number responses as a result of ${\text{M}}$ increasing at fixed ${\text{We }} = \, 0.{7},{\text{ R}} = {3},\chi_{{\text{Fe}_{3} {\text{O}}_{4} }} = 0.1,\,\chi_{{\text{Al}_{2} {\text{O}}_{3} }} = 0.05$.

**Figure 6:**
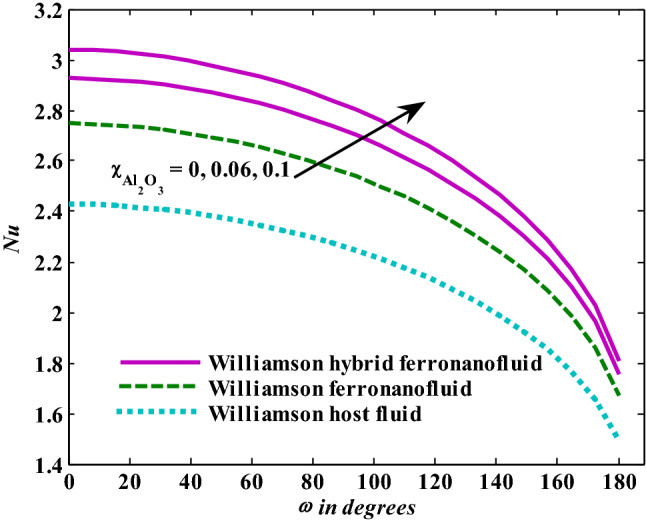
Nusselt number responses as a result of $\chi_{{\text{Al}_{2} {\text{O}}_{3} }}$ increasing at fixed ${\text{We }} = \, 0.{7},{\text{ M }} = \, 0.{8},{\text{ R }} = { 3}.$

Figure 3 depicts the Nusselt number’s trends as the Weissenberg number rises. The greater the Weissenberg number, the lower the heat transfer enhancement, and these tendencies were observed for base fluid, mono ferronanofluid, and hybrid ferronanofluid. Furthermore, the effect of the Weissenberg number on reduced heat transfer is stronger for hybrid ferronanofluid. Intuitively, it appears that impacts like Williamson's prevent fluid deformation, increasing viscosity of the fluid, reducing that the range of heat transfer. Figure 4 shows the tendencies of the Nusselt number when exposed to increased thermal radiation in the presence of the fixed effects of the other examined factors. The Nusselt number shows a clear increase when thermal radiation increases, which means an increase in the rate of energy transfer. It is normal for the heat transfer rate to increase as the radiation factor increases, because increasing it means releasing more energy into the fluid. Additionally, the effect of thermal radiation on the mono and hybrid ferronanofluids is greater than that of the ordinary fluid. Figure 5 presents the variations in the Nusselt number under conditions of a growing magnetic factor and with fixed effects of the other factors under investigation. When the magnetic parameter goes up, the Nusselt number obviously decays. This trend is expected from the Nusselt number because the increase in magnetic field strength leads to a slowdown in fluid velocity due to the stimulation of the formation of the Lorentz force and thus a decrease in the rate of energy transfer. Figure 6 shows the reaction of the Nusselt number when affected by increasing the volume fraction of aluminum oxide nanoparticles with fixation of the other parameters examined. It is evident that the Nusselt number increases with an increase in the volume fraction of aluminum oxide nanoparticles. This is because the increase in the volume fraction of aluminum oxide nanoparticles makes the thermal conductivity of the host fluid better.

**Figure 7:**
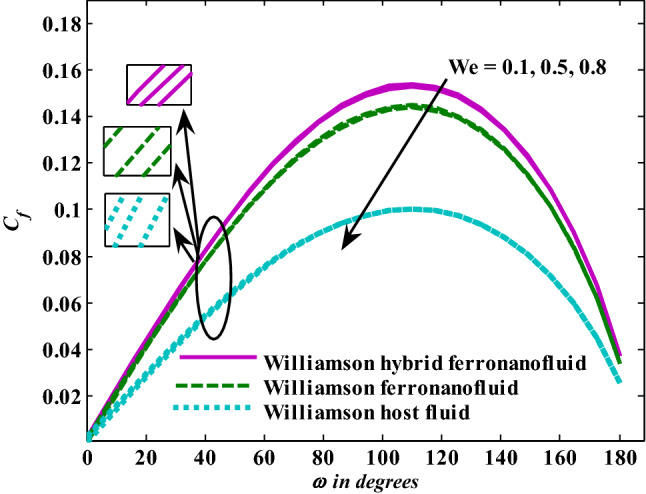
Skin friction responses as a result of ${\text{We}}$ increasing at fixed ${\text{R = 3,}}\,{\text{M = 0}}{.8},\,\chi_{{\text{Fe}_{3} {\text{O}}_{4} }} = 0.1,\,\chi_{{\text{Al}_{2} {\text{O}}_{3} }} = 0.05$.

**Figure 8:**
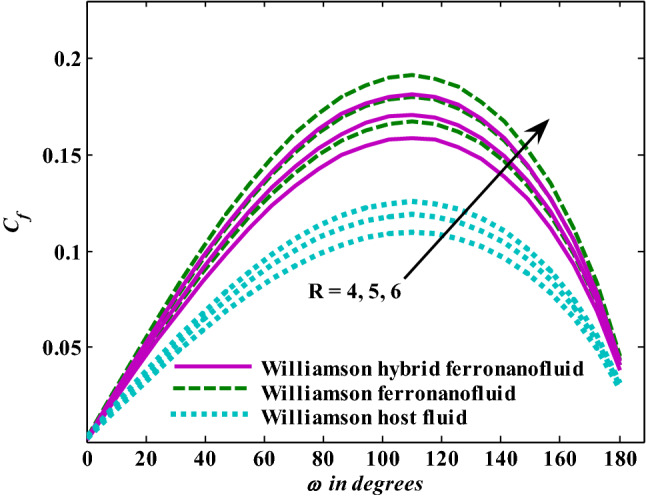
Skin friction responses as a result of ${\text{R}}$ increasing at fixed ${\text{We }} = \, 0.{7},{\text{ M }} = \, 0.{8}, \, \chi_{{\text{Fe}_{3} {\text{O}}_{4} }} = 0.1,\,\chi_{{\text{Al}_{2} {\text{O}}_{3} }} = 0.05$.

**Figure 9:**
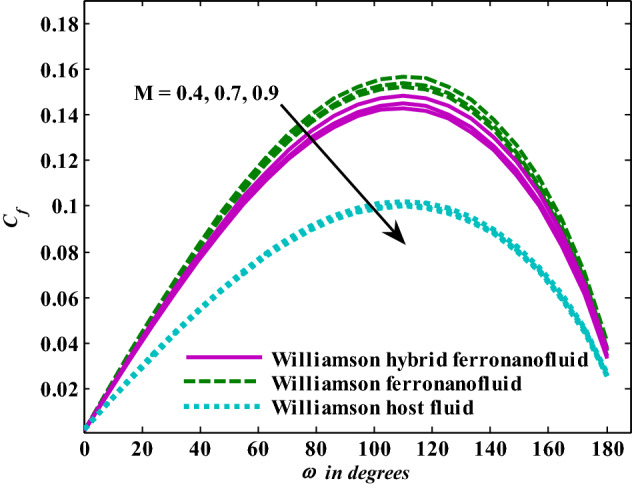
Skin friction responses as a result of M increasing at fixed ${\text{R}} = {3},{\text{ We }} = \, 0.{7}, \, \chi_{{\text{Fe}_{3} {\text{O}}_{4} }} = 0.1,\,\chi_{{\text{Al}_{2} {\text{O}}_{3} }} = 0.05$.

**Figure 10:**
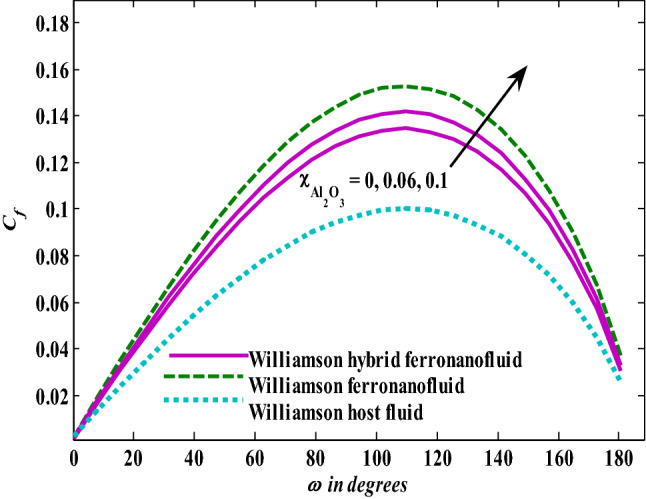
Skin friction responses as a result of $\chi_{{{\text{Al}}_{{2}} {\text{O}}_{{3}} }}$ increasing at fixed ${\text{We }} = \, 0.{7},{\text{ M }} = \, 0.{8},{\text{ R}} = {3}$.

**Figure 11:**
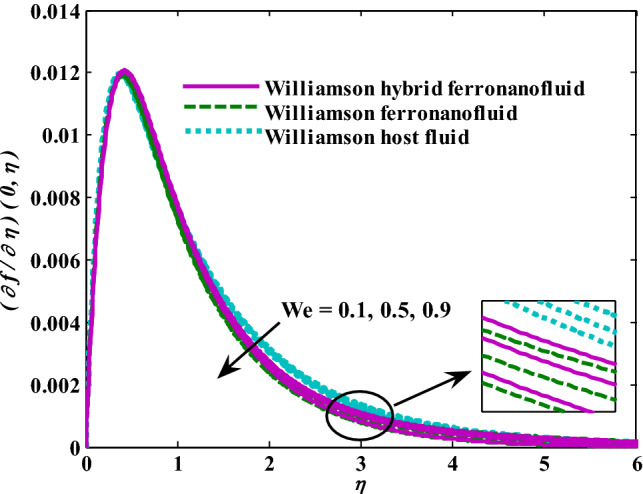
Velocity responses as a result of We increasing at fixed ${\text{R}} = {3},{\text{ M }} = \, 0.{8}, \, \chi_{{\text{Fe}_{3} {\text{O}}_{4} }} = 0.1,\,\chi_{{\text{Al}_{2} {\text{O}}_{3} }} = 0.05$.

**Figure 12:**
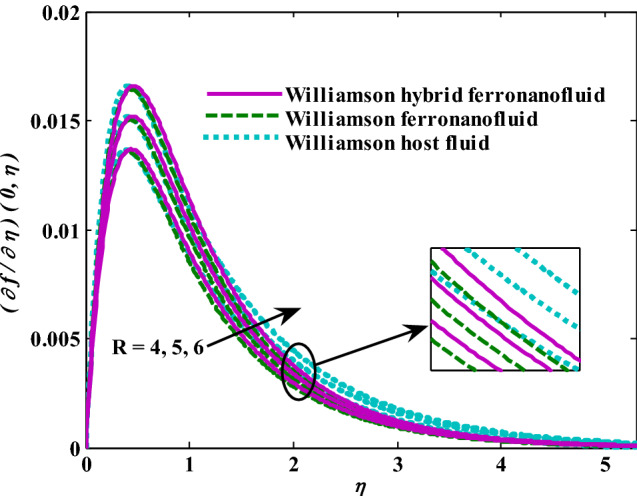
Velocity responses as a result of R increasing at fixed ${\text{We }} = \, 0.{7},{\text{ M }} = \, 0.{8}, \, \chi_{{\text{Fe}_{3} {\text{O}}_{4} }} = 0.1,\,\chi_{{\text{Al}_{2} {\text{O}}_{3} }} = 0.05$.

**Figure 13:**
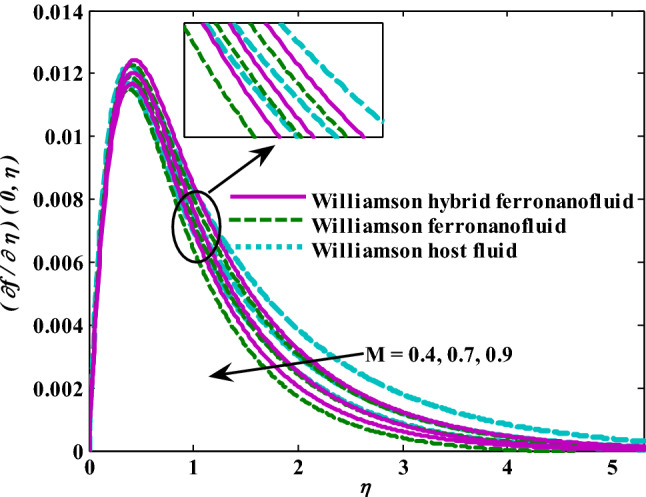
Velocity responses as a result of M increasing at fixed ${\text{We }} = \, 0.{7},{\text{ R }} = { 3},\chi_{{\text{Fe}_{3} {\text{O}}_{4} }} = 0.1,\,\chi_{{\text{Al}_{2} {\text{O}}_{3} }} = 0.05$.

**Figure 14:**
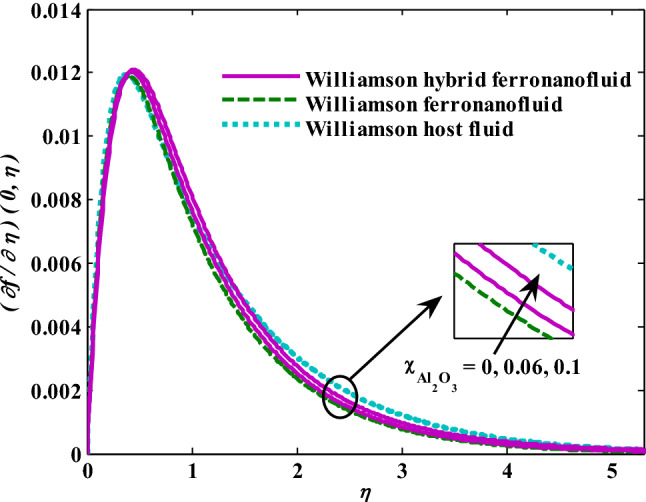
Velocity responses as a result of $\chi_{{{\text{Al}}_{{2}} {\text{O}}_{{3}} }}$ increasing at fixed ${\text{We }} = \, 0.{7},{\text{ M }} = \, 0.{8},{\text{ R }} = { 3}$.

**Figure 15:**
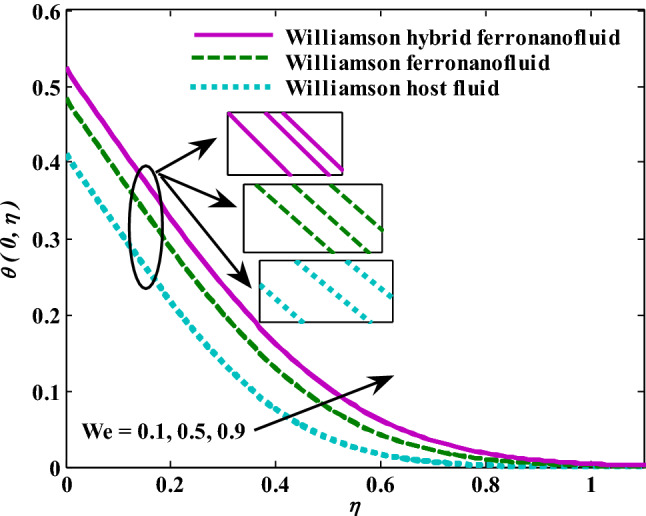
Temperature responses as a result of We increasing at fixed ${\text{R}} = {3},{\text{ M }} = \, 0.{8},\chi_{{\text{Fe}_{3} {\text{O}}_{4} }} = 0.1,\,\chi_{{\text{Al}_{2} {\text{O}}_{3} }} = 0.05$.

**Figure 16:**
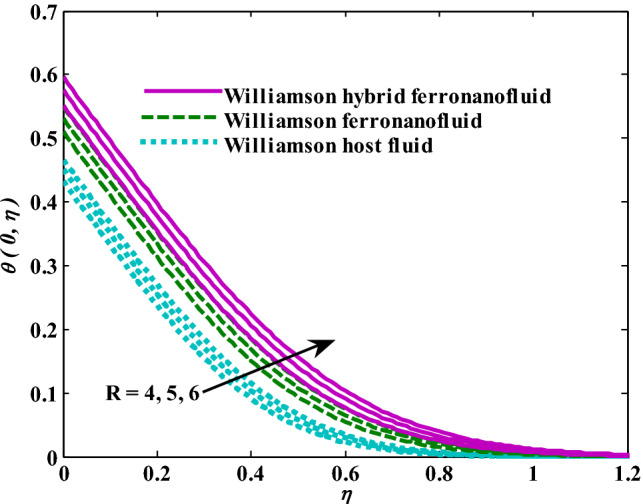
Temperature responses as a result of R increasing at fixed ${\text{We }} = \, 0.{7},{\text{ M }} = \, 0.{8},\chi_{{\text{Fe}_{3} {\text{O}}_{4} }} = 0.1,\,\chi_{{\text{Al}_{2} {\text{O}}_{3} }} = 0.05$.

**Figure 17:**
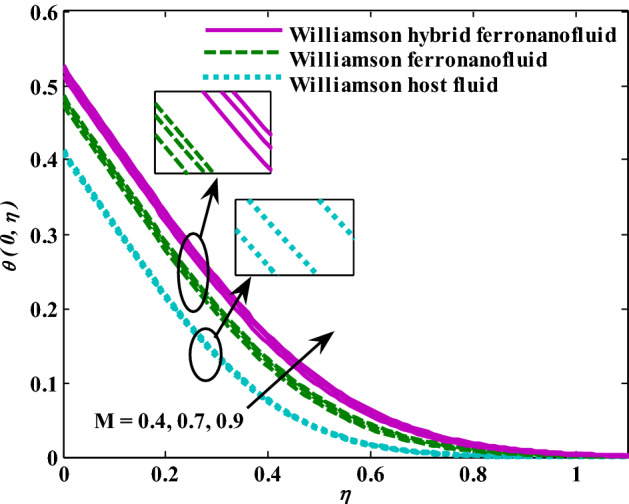
Temperature responses as a result of M increasing at fixed ${\text{We }} = \, 0.{7},{\text{ R}} = { 3},\chi_{{\text{Fe}_{3} {\text{O}}_{4} }} = 0.1,\,\chi_{{\text{Al}_{2} {\text{O}}_{3} }} = 0.05$.

**Figure 18:**
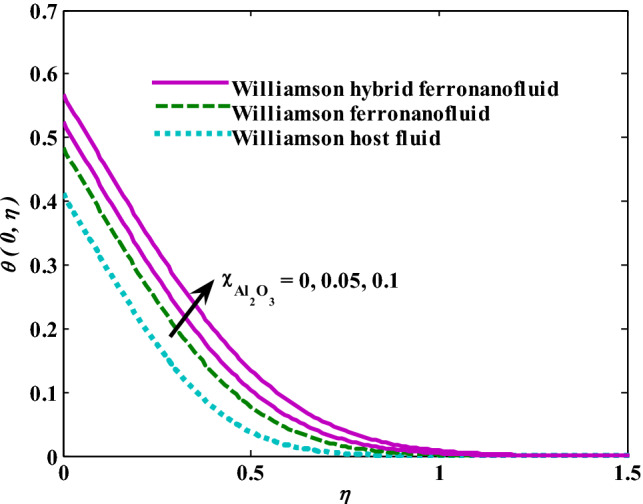
Temperature responses as a result of $\chi_{{{\text{Al}}_{{2}} {\text{O}}_{{3}} }}$ increasing at fixed ${\text{We }} = \, 0.{7},{\text{ M }} = \, 0.{8},{\text{ R}} = { 3}$.

Moreover, increasing the volume fraction factor by a few values increases the buoyancy forces and, of course, enhances the rate of energy transfer. Figures 3, 4, 5 and 6 show that the hybrid Williamson nanofluid has the highest energy transfer rate, followed by the mono-Williamson nanofluid, and the host fluid (engine-oil) has the lowest energy transfer rate. Figure 7 illustrates the relationship between the Weissenberg number and skin friction. It is noted that the coefficient of skin friction is negatively affected by the rising values of the Weissenberg number. An improvement in the relaxation time is associated with an increase in the values of the Weissenberg number, which in turn lowers the drag forces. In Fig. 8, the direct response to drag forces is shown with an increase in thermal radiation. Thermal radiation releases more energy in the fluid, which increases the collision of particles and thus increases the coefficient of skin friction. Figure 9 shows how the increase in the strength of the magnetic field affects the coefficient of skin friction. It is clear that this increase in the strength of the magnetic field reduces the drag forces brought on by the curbing that occurs in the fluid velocity due to the formation of the Lorentz force. Figure 10 shows how skin friction responds when the volume fraction values of aluminum oxide increase. It is clear that the reaction of the coefficient of friction is reversed as the coefficient of friction decreases with the increase in factor $\chi_{{{\text{Al}}_{{2}} {\text{O}}_{{3}} }}$. Physically, increasing the volume fraction factor increases the fluid velocity, which in turn raises frictional forces. The effects of increasing the values of the Weissenberg number on the velocity are depicted in Fig. 11. As the Weissenberg number values go up, the viscous forces get stronger, which slows the velocity of the fluid. Figure 12 exemplifies the effect of the radiation parameter on the distribution of fluid velocity. There is an obvious enhancement in the velocity when the radiation parameter grows. This trend could be explained by the fact that as the radiation rate increases, more energy is released into the liquid, which naturally enhances the velocity. Figure 13 illustrates how growth in magnetic parameter values impacts the velocity profile. A significant decrease in fluid velocity when the magnetic parameter is increased. It is well known that an increase in the intensity of the magnetic current stimulates the formation of the Lorentz forces, which in turn restrain the movement of the fluid. The direct relationship between the velocity and the volume fraction of aluminum oxide nanoparticles is clearly shown in Fig. 14. A higher volume fraction of aluminum oxide nanoparticles leads to an improvement in velocity profiles due to an increase in the thermal conductivity of the host fluid. It is worth mentioning here that the addition of nanoparticles improves buoyancy forces while at the same time improving viscous forces. Small values of nanoparticle volume fraction, ranging from 0.0 to 0.2, were used in previous studies, and these small values of nanoparticle volume fraction make the enhancement of the buoyancy force greater than the increase of the viscous forces, thus increasing the fluid's velocity. As for Fig. 15, it shows the changes in the temperature profiles when the Weissenberg number values rise. The temperature of the fluid increases with the increase in the values of the Weissenberg number, and this is, of course, due to the increase in the relaxation time. In Fig. 16, the positive effects of the thermal radiation coefficient on the temperature profiles are shown. As we mentioned earlier, increasing the thermal radiation factor means more energy is liberated inside the fluid, and this raises its temperature. Figure 17 shows how the temperature profiles are affected by increasing the magnetic parameter. It is clear that the increase in the magnetic parameter is accompanied by an increase in the temperature of the liquid. Increasing the magnetic parameter induces the formation of Lorentz forces. This force produces a kind of friction that has an impact on the flow and eventually raises the temperature as a result of the additional heat energy that this friction produces. Figure 18 presents the positive relationship between the fluid temperature and the volume fraction of aluminum oxide nanoparticles. Increasing the volume fraction of aluminum oxide improves the thermal conductivity of the host fluid, which increases the rate of energy transfer and thus increases the temperature. Finally, Figs. 11, 12, 13, 14, 15, 16, 17 and 18 show that the hybrid nanofluid is superior in terms of speed and temperature to the mono nanofluid and the host fluid in the boundary layer region, making it the center of interest in many applications.

## Conclusion

A careful analysis was executed of the natural convection flow of Williamson hybrid ferronanofluid affected by thermal radiation and magnetic field. Numerical outcomes for local skin friction and local Nusselt number, as well as temperature and velocity, were obtained using the Keller box technique. They satisfy the accuracy of current numerical solutions gained for the problem of free convection boundary layer flow in Williamson hybrid ferronanofluid. The following are the key findings from the current analysis:

1. Increasing the magnetization parameter or Weissenberg number suppresses the rate of energy transfer, while increasing the thermal radiation parameter or the volume fraction of hybrid nanoparticles improves it.
2. There is an inverse relationship between the drag forces and the Weissenberg number or the parameter of the magnetic field or the volume fraction of nanoparticles, while there is a direct relationship between the drag force and the parameter of thermal radiation.
3. The fluid’s velocity increases when the thermal radiation parameter or the volume fraction of a nanoparticle is increased, but it decreases when the magnetic radiation parameter or the Weissenberg number is increased.
4. Temperature is an increasing function of all the examined factors in this analysis.

The same issue is expected to be investigated in future work using other mathematical models, such as the Casson model, and it can also extend to include ternary hybrid nanofluids.

## Data Availability

The datasets used and/or analysed during the current study available from the corresponding author on reasonable request.

## List of symbols

*a*: Radius of cylindrical shape
*Bo*: Magnetic field strength
*Cf*: Skin friction coefficient
(*Cp*): Heat capacity
$f(\omega ,\eta )$: Dimensionless stream function
$$g$$: Gravity vector
_*Gr*_: Grashof number
$$\sigma$$: Electrical conductivity
*k*_*f*_: Thermal conductivity
_M_: Magnetic parameter
*Nu*: Nusselt Number
*P*: Fluid pressure
Pr: Prandtl number
$Q_{R}$: Rosseland diffusion approximation
Re: Reynold number
*T*: Temperature of the fluid
*T∞*: Ambient temperature
$\omega$: Component of velocity
*F*: Host liquid
M: Magnetic parameter
*U∞*: Uniform free stream
*η*: Component of velocity
*vf*: Kinematic viscosity of host liquid

## Greek symbols

$\alpha$: Thermal diffusivity coefficient
$\beta$: Thermal expansion of host liquid
$$\Gamma$$: Time constant
$\theta$: Temperature of nanoliquid
$$\kappa$$: Vortex viscosity
$\lambda$: Extra stress tensor
$$\mu$$: Dynamic viscosity
$$\rho$$: Density
$\phi$: Spin gradient viscosity
$\chi$: Nano-solid volume fraction
$$\psi$$: Stream transformation

## Subscript

*Hnf*: Hybrid nanoliquid
*nf*: Mono nanoliquid
*s*: Nanosolid
